# Extra-Axial Cerebellopontine Angle Low Grade Glioma – Unusual Occurrence in a 43-year-old Woman

**DOI:** 10.12669/pjms.41.13(PINS-NNOS).13492

**Published:** 2025-12

**Authors:** Zubair Mustafa Khan, Muhammad Jamil, Sameed Safdar Sheikh, Sumira Kiran

**Affiliations:** 1 Zubair Mustafa khan, MBBS, FCPS, Punjab Institute of Neurosciences, Lahore, Pakistan; 2 Muhammad Jamil, MBBS, Punjab Institute of Neurosciences, Lahore, Pakistan; 3 Sameed Safdar Sheikh, MBBS, Punjab Institute of Neurosciences, Lahore, Pakistan; 4 Sumira Kiran, MBBS, MS, Punjab Institute of Neurosciences, Lahore, Pakistan

**Keywords:** Cerebellopontine angle tumor, Glioma, Internal auditory canal, Oncology, Pakistan

## Abstract

Cerebellopontine angle (CPA) is an anatomical space of posterior cranial fossa bounded by tentorium superiorly, pons and cerebellum posteriorly and temporal bone anterolaterally. Glial cell tumors are located frequently in the cerebral hemispheres, while vestibular Schwannoma, meningioma and epidermoid cysts frequently affect the CPA. Low-grade gliomas (LGGs) presenting as a space occupying lesion (SOL) in cerebellopontine angle (CPA) is an extremely rare occurrence. This study reports a 43-year-old female, with no comorbidities, who presented with the chief complaints of headache and walking difficulty for six weeks, associated with left-sided facial numbness and hearing impairment. Neuro-imaging revealed left CPA SOL, which was excised via retrosigmoid craniectomy. Pathological work up was suggestive of diffuse low-grade glioma. The development of atypical SOLs, like glioma in this case, should be considered in the differential diagnosis of cerebellopontine angle tumors. The standard treatment is maximum safe resection in case of LGGs with serial neuroimaging and adjuvant management.

## INTRODUCTION

Glial cell tumors with an incidence rate of around six per 100,000 population in the United States are the most common primary CNS tumors in adults.^1^ Primary infratentorial gliomas are uncommon in adult patients and are found in approximately 7.53% (336/4,462 cases) of patients according to National Cancer Institute’s Surveillance, Epidemiology, and End Results (SEER) program.^2^ Cerebellopontine angle (CPA) low-grade gliomas are extremely rare and represent exophytic lesions from the cerebellar peduncle.^3^ Primary glioma in CPA arises from the root entry zone of the cranial nerves in the CPA.^1^ Signs and symptoms are mostly due to compression of the fifth, seventh, and eighth cranial nerves and the lateral aspect of the pons and cerebellum.

Tumors of neuroepithelial origin like pilocytic astrocytomas, brainstem gliomas, medulloblastomas, or ependymomas are the most commonly observed tumors in the posterior fossa in childhood.^4^ CPA is frequently known to be affected by acoustic Schwannomas, meningiomas and epidermoid cysts in adults.^5^,^6^ Handful cases of pilocytic astrocytoma and glioblastoma in CPA are documented in existing scientific literature.^5^,^6^ However, the occurrence of CPA low-grade glioma (LGG) is exceedingly rare.

## CASE PRESENTATION

**Fig.1:**
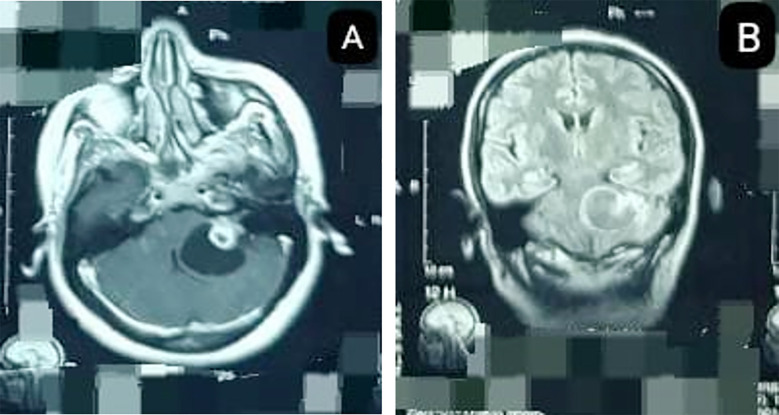
Contrast-enhanced MRI (CE-MRI) brain (A) axial & (B) coronal view showing heterogeneous left CP Angle SOL.

A 43-year-old female, with no comorbidities, presented in the outpatient clinic of Punjab Institute of Neurosciences in January 2025 with the chief complaints of headache and walking difficulty for six weeks, associated with left-sided facial numbness and hearing impairment. There was no significant past medical or surgical history. Her higher mental functions were intact. There was left-sided facial numbness with House Brackmann Grade-II weakness and sensorineural hearing loss on the left side. The rest of the cranial nerves were intact. There was ataxia, nystagmus on the left side and monoparesis of the left lower limb. The patient went through a series of diagnostic investigations. Non-contrast enhanced computed tomography (NCE-CT) brain showed a hypodense space-occupying lesion in the left cerebellopontine angle with a well-defined cystic component causing mass effect and compressing the 4^th^ ventricle. CT brain was not suggestive of any calcifications. Contrast-enhanced magnetic resonance imaging (CE-MRI) of the brain showed a well-defined, rounded, thin-walled cystic lesion with an avidly enhancing solid mural nodule of size 32 x 32 x 24 mm with minimal peripheral vasogenic edema (Fig.1). The mural nodule appeared hypointense on T1 and hyperintense on T2 with no significant diffusion restriction on DWI and ADC mapping. The lesion was partially effacing the fourth ventricle, with no hydrocephalus, and left-sided CP angle cistern and extending into the internal acoustic meatus. A hemangioblastoma or pilocytic astrocytoma were the differentials under consideration.

**Fig.2:**
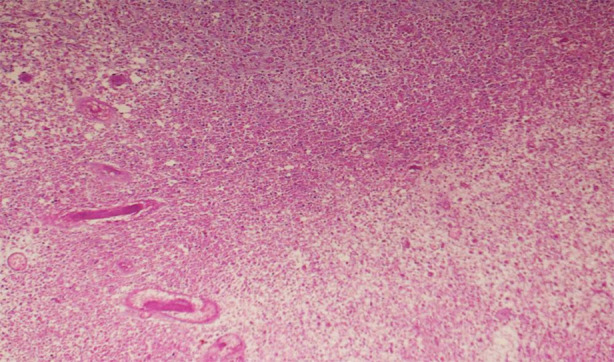
Histopathology under light microscope with hematoxylin and eosin (H & E) stains. Variable hypercellularity with individual tumor cells infiltrating the brain parenchyma. Diffuse infiltration results in ill-defined margin with adjacent brain tissue. Features suggestive of diffuse low-grade glioma.

Operative intervention for the tumor was planned under general anesthesia, unfortunately in the absence of intraoperative neuromonitoring due to limited resources. The left CP angle SOL was excised via retrosigmoid craniectomy. Gross total resection was achieved with minimal blood loss. Intraoperative findings showed a pial based, yellowish grey, soft, easily suckable, moderately vascular left-sided CP angle SOL having both cystic and solid components. The tumor was in close proximity to the facial nerve but was not adherent to any nerve. The surgery remained uneventful. Her postoperative recovery was smooth and discharged on the second postoperative day. At two weeks’ follow-up, the patient had persistent left-sided sensorineural hearing loss with no new neurological deficit. There was no surgical site infection, and she and her family were reassured. The patient was lost to follow up after she was referred to a neighboring hospital for consultation with regard to adjuvant therapy.

The histopathology report suggested a diagnosis of diffuse low-grade glioma grade 1/2 according to WHO CNS tumor classification of 2021. On microscopy, sections revealed a glial neoplasm comprising of oval to spindle glial cells with mild atypia with the absence of Rosenthal fibers, thus ruling out pilocytic astrocytoma. No significant mitotic activity, necrosis or microvascular proliferation were seen. Immunostains used were: GFAP: positive, Olig2: positive. IDH1: equivocal, ATRX: retained expression. A polymerase chain reaction (PCR) test was required to differentiate between IDH1 and IDH2 mutant, but this could not be performed due to the patient’s refusal for further testing.

## DISCUSSION

Gliomas are mostly found intra-axially and in rare cases can occur extra-axially.^7^ The origin of extra-axial gliomas remains unknown, however neural crest cells are considered the most likely cause.^7^ Many glioma cells are undifferentiated and do not express any specific cell markers, except nestin, which is also a marker for neural crest cells.^7^ Additionally, neural crest cells show other similar findings to gliomas such as high motility and association with white matter tracts.^7^ Myelin may play a protective role against glioma invasion and its destruction can thus lead to tumor invasiveness.^8^ LGGs tend not to metastasize, however one factor that seems to play a role in metastasis of high grade gliomas, such as glioblastoma multiforme, is the aggressiveness of the cell type.^9^

According to the Central Brain Tumor Registry of the United States (CBTRUS) 2016-2020 report, gliomas (malignant and non-malignant) accounted for 26.3% of all brain and other central nervous system tumors overall, and 51.1% of brain and CNS tumors in children aged 0-14 years, with most of them (62.2%) occurring in the supratentorial region (frontal, temporal, parietal, and occipital lobes).^1^ LGGs consist of grade I and II gliomas as per the World Health Organization’s classification and represent two-thirds of gliomas in adolescents, while the remaining one-third consist of grades III and IV.^10^ They are the most common childhood brain tumors and represent 30% of all brain tumors in children.^11^ In the United States, the annual incidence of pediatric LGGs is 1.3-2.1 per 100,000, whereas in adults it is higher with an annual incidence of 9.1-12.5 cases per 100,000. They occur most frequently in the second to fourth decades of life and over 90% of them are located in the supratentorial region.^10^,^12^,^13^ In our case the patient was aged 43 years, mimicking the peak age distribution for gliomas, and was located in the cerebellopontine angle.

The most common tumor found in the cerebellopontine angle is vestibular schwannoma (80-90% of cerebellopontine angle tumors) and the second most common is meningioma (10% of CPA tumors).^4^,^6^ The presence of glial cell tumors in the cerebellopontine angle is rare, representing less than one percent of cerebellopontine angle tumors.^14^,^15^

The cerebellopontine angle contains cranial nerves V, VII, and VIII and most patients with cerebellopontine angle tumors present with symptoms involving these nerves such as facial numbness/pain, facial muscle weakness, and hearing loss, as well as difficulty walking (ataxia), muscle weakness, and headache.^4^,^14^ Wu et al. also described symptoms of difficulty speaking and swallowing in patients with glioblastoma multiforme of the cerebellopontine angle.^16^ According to Adib et al’s review of the literature most patients presented with symptoms lasting 1-3 months, and a few had symptoms lasting for many months.^15^ Our case presented with complaints of headache, difficulty walking, left sided facial numbness, and hearing impairment for six weeks. Similarly this case presentation in our patient was consistent with exophytic CPA pathology.

According to existing literature, LGGs show hypointensity on T1-weighted images, hyperintensity on T2-weighted images, and usually no contrast enhancement.^17^ These findings are non-specific and can be seen in vestibular schwannomas and malignant gliomas such as glioblastoma multiforme as well, thus making it difficult to distinguish LGGs from other tumors on these findings alone.^18^ The main differentiating feature between LGGs and high grade gliomas is the presence of contrast enhancement in high grade gliomas, although this is not highly specific either, since some high grade gliomas can lack contrast enhancement and some LGGs can exhibit it.^18^

Our case demonstrated a hypodense space-occupying lesion in the left cerebellopontine angle with well-defined, round, cystic component causing mass effect and compressing the 4^th^ ventricle on non-contrast enhanced CT. On contrast-enhanced magnetic resonance imaging (CE-MRI) a rounded, thin-walled cystic lesion with an avidly enhancing solid mural nodule with minimal peripheral vasogenic edema was seen in our case. The mural nodule appeared hypointense on T1 and hyperintense on T2 with no significant diffusion restriction on DWI and ADC mapping, which are consistent with glioma findings in the existing literature.^19^ Breshears et al. and Marak et al. suggested MR spectroscopy was helpful in differentiating malignant tumors from benign ones by showing decreased NAA peak with elevated creatinine and choline peak in malignant cases.^5^,^19^

According to a 19 year retrospective study of 554 adults with nonpilocytic LGGs, achieving maximally safe resection was associated with improved overall survival outcomes compared to subtotal resection and less aggressive surgical treatment.^13^ The mainstay of treatment remains surgical resection with postoperative radiotherapy being a potential treatment option in high-risk patients (deep tumors, size ≥ 5 cm, astrocytomas, receiving subtotal resection/biopsy only, and age > 40 years).^13^ In our case, gross total resection of the lesion was achieved via retrosigmoid craniectomy with no intraoperative complications, and no adjuvant therapy was administered.

The diagnosis of gliomas in the cerebellopontine angle remains challenging due to its very rare presentation as well as its nonspecific findings on imaging. Although our case presented with the typical findings on MRI, its presentation in the cerebellopontine angle is rare varied as detailed in the existing literature, thus making it difficult to diagnose on imaging. We conducted review of the literature using Advanced Search based on “Title” and the Boolean operator (AND) on PubMed from inception until August 20, 2025 yielding the following results for the following word combinations (Table-I). Out of these 41 studies, only one study resembled our case (Table-II).

**Table-I:** Results of review of the literature on PubMed using a combination of words.

Searches	Combination of words	Results on PubMed
Search 1	"cerebellopontine" AND "glioblastoma"	14
Search 2	"cerebellopontine" AND "glioma"	5
Search 3	"cerebellopontine" AND "astrocytoma"	13
Search 4	"cerebellopontine" AND "pilocytic"	9
Total		41

**Table-II:** Summary of the relevant case from review of the literature on PubMed using Search 2.

Study by	Title	Age & Gender	Radiology	Histopathology
Zhou et al.^20^ 2023	A case of primary glioma in the cerebellopontine angle - 2023	38y, M	MRI showing occupying lesion in the left CPA, suggesting an extra-axial benign mass	Tumor cells were positive for GFAP, p53, NF, S-100, while negative for IDH-1. The pathological diagnosis was adult-type diffuse glioma with WHO grade 2-3

Legend: M: male, y: years, MRI: magnetic resonance imaging, CPA: cerebellopontine angle, GFAP glial fibrillary acidic protein, WHO: World Health Organization.

## CONCLUSION

Low-grade glioma (LGG) of the cerebellopontine angle is extremely rare and its findings on neuro-imaging are nonspecific, making its preoperative diagnosis difficult, especially with regard to other common cerebellopontine angle tumors such as vestibular schwannoma and meningioma. As for other CPA SOLs, maximal safe resection with minimal damage to adjacent parenchyma and cranial nerves is the principal of surgery for a CPA-LGG as achieved in our patient.

### Author`s Contribution:

**ZMK:** Concept and design of the work, critical review of manuscript and supervision. **MJ, SSS:** Data acquisition and data interpretation, drafted the manuscript. **SK:** Data analysis, critical review of manuscript. All the authors have read and approved the final manuscript for the publication. All agreed to be accountable for all aspects of the work related to accuracy or integrity.
